# Transcript level coordination of carbon pathways during silicon starvation‐induced lipid accumulation in the diatom *Thalassiosira pseudonana*

**DOI:** 10.1111/nph.13843

**Published:** 2016-02-04

**Authors:** Sarah R. Smith, Corine Glé, Raffaela M. Abbriano, Jesse C. Traller, Aubrey Davis, Emily Trentacoste, Maria Vernet, Andrew E. Allen, Mark Hildebrand

**Affiliations:** ^1^ Scripps Institution of OceanographyUC San Diego9500 Gilman DriveLa JollaCA92093USA; ^2^ J. Craig Venter Institute4120 Capricorn LaneLa JollaCA92037USA

**Keywords:** carbon metabolism, cell cycle, diatom, lipid metabolism, *Thalassiosira pseudonana*, transcriptomics

## Abstract

- Diatoms are one of the most productive and successful photosynthetic taxa on Earth and possess attributes such as rapid growth rates and production of lipids, making them candidate sources of renewable fuels. Despite their significance, few details of the mechanisms used to regulate growth and carbon metabolism are currently known, hindering metabolic engineering approaches to enhance productivity.
- To characterize the transcript level component of metabolic regulation, genome‐wide changes in transcript abundance were documented in the model diatom *Thalassiosira pseudonana* on a time‐course of silicon starvation. Growth, cell cycle progression, chloroplast replication, fatty acid composition, pigmentation, and photosynthetic parameters were characterized alongside lipid accumulation.
- Extensive coordination of large suites of genes was observed, highlighting the existence of clusters of coregulated genes as a key feature of global gene regulation in *T. pseudonana*. The identity of key enzymes for carbon metabolic pathway inputs (photosynthesis) and outputs (growth and storage) reveals these clusters are organized to synchronize these processes.
- Coordinated transcript level responses to silicon starvation are probably driven by signals linked to cell cycle progression and shifts in photophysiology. A mechanistic understanding of how this is accomplished will aid efforts to engineer metabolism for development of algal‐derived biofuels.

## Introduction

Diatoms are a diverse group of marine and aquatic photosynthetic microbial eukaryotes that form the base of oceanic food webs and are important in biogeochemical cycling of many elements (carbon (C); nitrogen (N); silicon (Si); iron (Fe)) globally. Additionally, many diatoms produce abundant lipids, making them candidates for the development of renewable biofuels from microalgae (Hildebrand *et al*., 2012; Levitan *et al*., 2014). They were the first group of marine phytoplankton with a full genome sequence available (Armbrust *et al*., 2004), and subsequently more representatives have been sequenced (Bowler *et al*., 2008; Lommer *et al*., 2012; http://www.jgi.doe.gov). The availability of genomic data and focused efforts to develop tools for genetic manipulation of diatoms has facilitated in‐depth investigations into these organisms (Poulsen & Kröger, 2005; Poulsen *et al*., 2006; Bozarth *et al*., 2009; Allen *et al*., 2011; Karas *et al*., 2015). However, while these data provide valuable insight into the evolutionary history and metabolic potential of these important organisms, there is still little known about the relative role of various regulatory processes on metabolism and cell function. Approaches grounded in functional genomics, such as quantitative global transcript profiling, can be used to characterize how organisms employ the information encoded in genomes to replicate and adapt to fluctuating environmental conditions.

Transcriptomics generally aims to characterize how mRNA‐level changes underlie a variety of physiological, metabolic, and developmental processes. Advances in high‐throughput sequencing have facilitated culture‐based transcriptomic studies in diatoms, providing insight into the adaptive response of these organisms to environmental change, such as high light (Park *et al*., 2010), carbon dioxide (Hennon *et al*., 2015), phosphorus stress (Dyhrman *et al*., 2012), nitrogen and silicon limitation and release (Mock *et al*., 2008; Sapriel *et al*., 2009; Hockin *et al*., 2012; Shrestha *et al*., 2012; Bender *et al*., 2014), iron starvation (Allen *et al*., 2008), and other stressors (Maheswari *et al*., 2010). A more complete understanding of the significance of transcriptional control of cellular processes is important for an improved understanding of the basic biology of diatoms and other microalgae, and has implications for environmental and biotechnological studies. For example, metabolic engineering to improve lipid productivity is widely considered to be an essential element of economic feasibility and commercialization of algae as a feedstock for renewable biofuels (Radakovits *et al*., 2010; Davis *et al*., 2011). Appropriate selection of targets for effective genetic engineering is required to accomplish these tasks.

Regulation of cellular function is complex and occurs at multiple levels and, as a result, transcriptomes alone are not necessarily predictive of a physiological or metabolic response. Metabolic flux is ultimately regulated by the activities of enzymes in an individual pathway, which are controlled by many factors, including protein abundance (governed by their relative rate of synthesis or degradation), substrate concentrations, allosteric interactions, and post‐translational modifications (Plaxton, 1996). Transcript abundance impacts overall enzyme activity through regulation of cellular potential to synthesize new proteins, but the rigor of the connection can vary. Conventionally, transcript level regulation has been considered to be more important during development or during long‐term adaptation (Plaxton, 1996). With an increasing number of studies utilizing advanced‐omic approaches, a deeper understanding of the significance of transcript level regulation of cellular metabolism and physiology is emerging in a variety of different organisms (Tu *et al*., 2005). In eukaryotic algae, the relationship between global transcript changes and metabolic and physiological shifts has been examined only in a limited context (Nymark *et al*., 2009; Chauton *et al*., 2012). Understanding the contribution of transcript level control to cellular metabolic responses would be advantageous for metabolic engineering approaches, as these typically involve artificially regulating mRNA levels through overexpression or knockdown techniques.

In diatoms, there are examples of genes that are transcriptionally responsive to specific environmental conditions, suggesting that changes in transcript abundance correlate with metabolic demand and physiological status. For example, the expression of silicon transporters is up‐regulated during silicon starvation in diatoms (Hildebrand *et al*., 1998), and genes in the photorespiratory pathway are up‐regulated during increased glycolate production (Parker *et al*., 2004). Genes such as LHCX1 and AUREOCHROME 1‐a in *Phaeodactylum tricornutum* are known to be light‐responsive (Bailleul *et al*., 2010; Costa *et al*., 2013). In the case of AUREOCHROME 1‐a, the specific mechanism by which light regulates cell division through the activity of dsCYC2 (a diatom‐specific cyclin) has been elucidated (Huysman *et al*., 2013). Alternatively, recent functional genomics studies in diatoms and other microalgae have shown that transcription of large suites of genes is coordinated with growth‐related processes such as chloroplast division, release from nitrogen and silicon starvation, cell wall synthesis, onset of cell division, and circadian shifts, suggesting that many genes may be under the control of master regulators (i.e. redox state, transcription factors) that choreograph genome‐wide transcription (Gillard *et al*., 2008; Monnier *et al*., 2010; Allen *et al*., 2011; Shrestha *et al*., 2012; Ashworth *et al*., 2013). Consequently, changes in transcript abundance can be interpreted as an adaptive response to specific environmental conditions, as a part of a coordinated regulatory program associated with growth, or a combination of both factors.

In many algae, including diatoms, starvation for essential nutrients (i.e. N, phosphorus) induces the formation of triacylglycerol‐rich lipid droplets coincident with an arrest in growth (Hu *et al*., 2008; Yu *et al*., 2009). Silicon is required for cell wall synthesis and growth in most diatoms, and silicon starvation also induces growth arrest and the formation of lipid droplets (Shifrin & Chisholm, 1981; Roessler, 1988; Traller & Hildebrand, 2013). In contrast to nitrogen starvation (Gasch *et al*., 2000; Hockin *et al*., 2012; Bender *et al*., 2014), silicon starvation has little effect on overall metabolic activities, making it a unique approach to distinguish between cell cycle arrest and secondary effects that arise during nutrient limitation (Darley & Volcani, 1969; Claquin *et al*., 2002).

We present a transcriptomic analysis of the response of *Thalassiosira pseudonana* during a time‐course of silicon limitation. To relate changes in transcript abundance to cellular processes, we also evaluated growth data (cell concentration, cell cycle progression), cellular composition (lipid concentrations, pigment concentrations), and photophysiology (carbon fixation, physiological fluorescence). The data provided insight into the cellular processes associated with carbon acquisition and assimilation by demonstrating that changes in transcript abundance of many carbon and energy metabolism genes are decoupled from shifts in metabolism and physiology and are additionally regulated by cell growth and division. A high degree of coordinated regulation of transcript abundances for genes involved in distinct photosynthetic and metabolic processes in different cellular compartments was documented. These clusters are sets of coordinately regulated genes under the putative control of as yet unknown master regulators such as metabolites, cellular redox state, or transcription factors (Ma *et al*., 2013). Despite a limited understanding of the specific mechanisms that regulate coexpression modules, the high degree of transcriptome orchestration demonstrates that *T. pseudonana* utilizes a regulatory hierarchy to integrate transcript abundance of many genes involved in key energetic and carbon sources during metabolic shifts.

## Materials and Methods

Axenic 8 l cultures of *Thalassiosira pseudonana* (Hustedt) Hasle et Heimdal (CCMP1335) were grown in artificial seawater medium (NEPC; http://www3.botany.ubc.ca/cccm/NEPCC/esaw.html) at 18°C under continuous light (150 μmol m^−2^ s^−1^) to a concentration of *c*. 1 × 10^6^ cells ml^−1^, harvested by centrifugation for 12 min at 3100 ***g***, and then placed in 8 l of silicon‐free (Si–) medium in a polycarbonate bottle at a concentration of *c*. 5 × 10^5^ cells ml^−1^ in experiments Si– #1 to Si– #8, and 1 × 10^6^ cells ml^−1^ in experiments Si– #9 and Si– #10. Cultures were stirred and bubbled with air under continuous light, and sampled at 0, 4, 8, 12, 18 and 24 h following inoculation into silicic acid‐free medium to evaluate several cellular parameters. Parameters investigated were genome‐wide transcript abundance (using both microarrays and RNA‐Seq), cell cycle progression and growth, lipid content (lipophilic dyes and fatty acid methyl ester analysis), pigment concentrations, shifts in photophysiology (photosynthesis–irradiance (*P*–*I*) curves and fast repetition rate fluorometry), and chloroplast features using imaging flow cytometry. Details of these methods in addition to which biochemical and physiological parameters were evaluated for a given experiment can be found in the supporting information (Supporting Information Methods S1; Table S1; Folch *et al*., 1957; Platt *et al*., 1975; Lewis & Smith, 1983; Benjamini & Hochberg, 1995; Kolber *et al*., 1998; Arrigo *et al*., 1999; Hildebrand & Dahlin, 2000; Zapata *et al*., 2000; Dodds *et al*., 2005; Shrestha *et al*., 2012; Kim *et al*., 2013; Love *et al*., 2014).

Transcriptomes were analyzed from experiments Si– #3, Si– #9, and Si– #10. RNA from experiment Si– #3 was processed for hybridization to an Affymetrix GeneChip whole‐genome tiling array (in technical duplicate, Methods S1) while RNA from Si– #9 and Si– #10 was processed for Illumina‐based RNA‐Seq. Statistical analyses for significance are detailed in Methods S1. To quantify the replication of the response in genome tiling and RNA‐Seq experiments, the Pearson correlation coefficient (PCC) between the response of a given gene from the microarray and RNA‐Seq data was determined using core signal fluorescence intensities (from the microarray) and the DESeq2 normalized counts (from RNA‐Seq). The distribution of PCCs shows a generally good agreement, indicating an overall correlation between experiments (Fig. S1). Both microarray data and RNA‐Seq data are plotted throughout as fold‐change (log_2_) relative to the 0 h time point (Fig. S1). For RNA‐Seq, no organellar genome data are available for comparison because of the poly‐A purification step during sequencing library preparation.

Transcript abundance patterns of both microarray and RNA‐Seq data were clustered using *k*‐means clustering with log_2_ fold‐change values relative to *t* = 0 for both datasets (Genesis 1.7.6, Sturn *et al*., 2002). A range of cluster sizes was tested (in increments of five from five to 50, and 10 from 50 to 100). Average within‐cluster variance of actual data was lower than that of a randomized dataset at 50 clusters, which was chosen as an acceptable threshold. Centroid values were then reclustered using hierarchical clustering (agglomeration set to average, Genesis 1.7.6; Sturn *et al*., 2002). The numbers of genes annotated with EuKaryotic Orthologous Groups (KOG) classes were tabulated for each cluster. As both clusters and KOG classes have different numbers of genes attributed to them, a functional enrichment index (FEI) was calculated to determine if clusters were disproportionately enriched in any functional categories:

$$\text{FEI}=(G_{\mathrm{clusterclass}}/\left(G_{\mathrm{cluster}}\times G_{\mathrm{class}}\right))\times 1000.$$

where *G*_clusterclass_ is the number of genes assigned to both a given expression cluster and KOG class, *G*_cluster_ is the total number of genes represented in that cluster, and *G*_class_ is the total number of genes in a given KOG class.

## Results

### Silicon starvation‐induced physiological shifts

**Figure 1:**
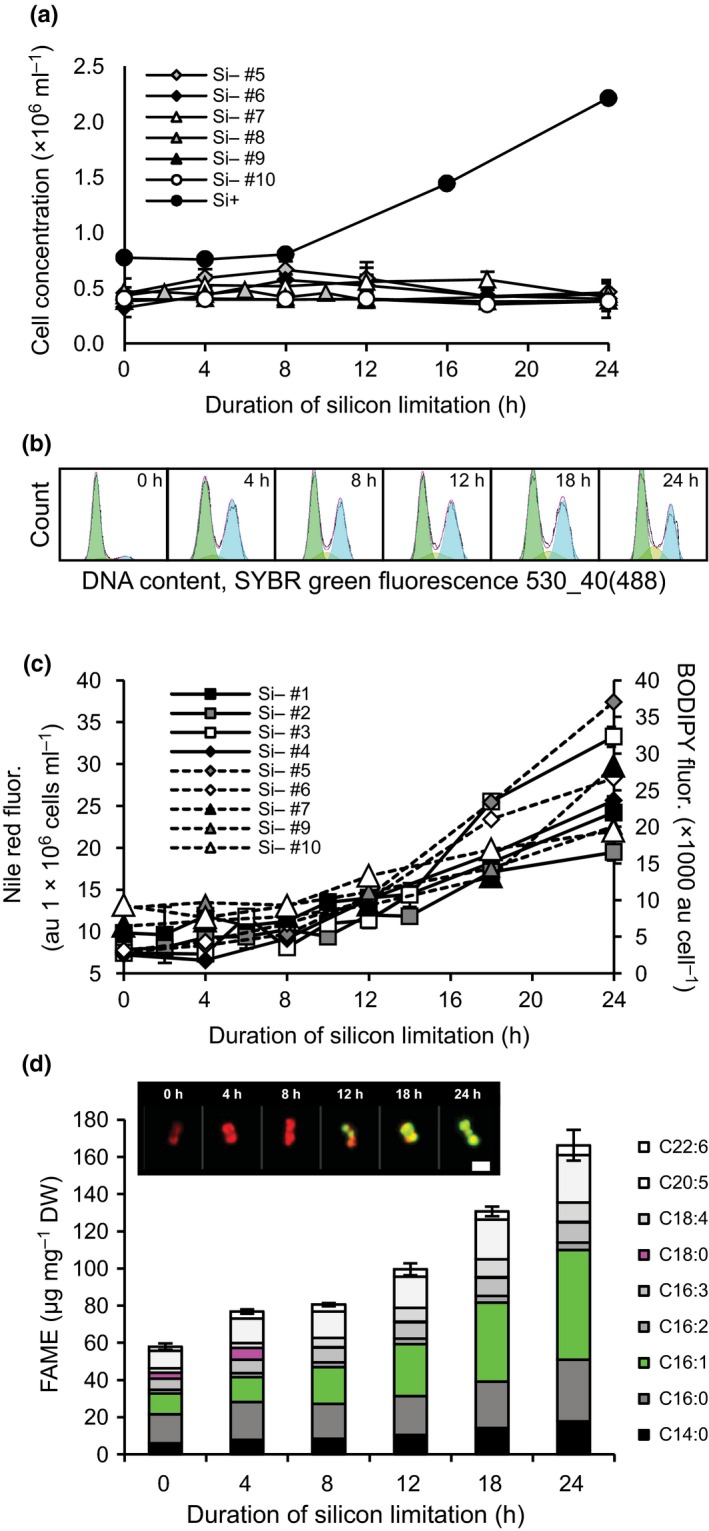
(a–d) Growth (a), cell cycle (b), and lipid content (c, d) in *Thalassiosira pseudonana* during silicon starvation. Cell concentrations over time (a) in several experiments after transfer to silicon‐free medium shown with a silicon‐replete control (Si+). Representative cell cycle histogram (b, from Si– #5). In each histogram, the left peak shows cells in the G1 phase of the cell cycle, and the right peak shows G2 + M cells. Lipid content over time determined fluorometrically using Nile Red (solid lines) and BODIPY (dashed lines) in the different silicon limitation experiments (numbered) (c) and average fatty acid methyl ester (FAME) profile (d) from Si– #5, Si– #9, and Si– #10. Error bars show ± SD for total FAME content between experiments. Inset micrographs show Chl (red) and BODIPY‐stained lipid droplets (green) in a cell representing the average of the population at each experimental time point (bar, 3 μm). au, arbitrary units.

Cell division in *T. pseudonana* was arrested immediately in silicon‐free medium, while partial progression through the cell cycle continued until *c*. 40% of the population was in the G2 + M phase at 4 h (Fig. 1a,b). Both the silicon transporters SIT1 (Thaps3_268895) and SIT2 (Thaps3_41392) were up‐regulated at the transcript level by 4 h and remained high throughout the time‐course, confirming silicon limitation (Table S2; Hildebrand *et al*., 1998; Thamatrakoln & Hildebrand, 2007). Cellular lipid concentrations (determined fluorometrically) induced reproducibly between 8 and 12 h of silicon starvation, which overall corresponded to a nearly threefold increase in cellular fatty acid methyl ester (FAME) concentrations (Fig. 1c,d). After 12 h of silicon starvation, lipid droplets could be detected with BODIPY staining (Fig. 1d, inset), and were coincident with a shift in the dominant FAME from palmitic acid (C16:0) to palmitoleic acid (C16:1), the latter of which is a known major constituent of diatom triacylglycerol (TAG; Fig. 1d; Yu *et al*., 2009).

**Figure 2:**
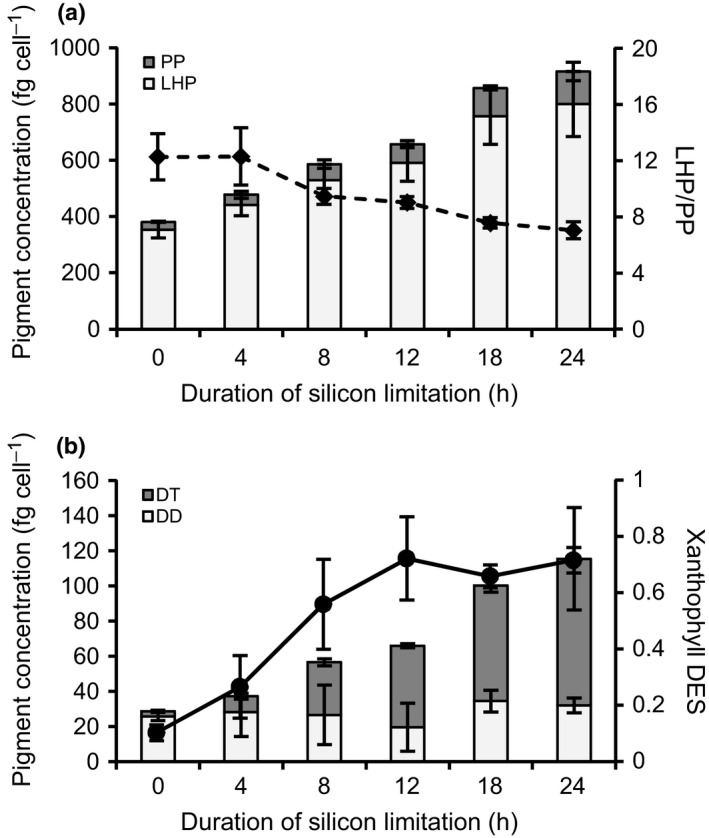
Cellular pigmentation changes during silicon starvation in *Thalassiosira pseudonana*. (a) Pigment concentrations per cell shown as light‐harvesting pigments (LHP; Chl*a*, Chl*c*, fucoxanthin) and photoprotective pigments (PP; diatoxanthin and diadinoxanthin) are plotted and represent the average of technical duplicates from Si– #5, Si– #6, and Si– #7. The average ratio of light‐harvesting pigments for Si– #5, Si– #6, and Si– #7 is plotted (dashed line). Error bars show ± SD between experiments. Pigment concentrations of xanthophyll pigments diatoxanthin (DT) and diadinoxanthin (DD) (b) are plotted and represent the average of technical duplicates from Si– #5, Si– #6, and Si– #7. The xanthophyll de‐epoxidation state (DES) is plotted and represents the average of technical duplicates from Si– #5, Si– #6, and Si– #7. Error bars show standard deviation between experiments.

In addition to TAG increases, total cellular pigment concentrations increased 2.4‐ to 2.7‐fold after 24 h of silicon starvation in several experiments (Si– #5, Si– #6, Si– #7; Fig. 2a). There was a disproportionate increase in photoprotective xanthophyll pigments (diatoxanthin and diadinoxanthin, 3.3‐ to 5.1‐fold per cell) relative to light harvesting pigments (Chl*a*, Chl*c*, and fucoxanthin, 2.1‐ to 2.4‐fold per cell, Fig. 2). The de‐epoxidation state (DES) of the xanthophyll pool also increased, indicating enhanced dissipation of absorbed light energy through nonphotochemical quenching (NPQ; Fig. 2b). Somewhat paradoxically, silicon‐starved *T. pseudonana* increases cellular pigment concentrations while simultaneously dissipating light energy through increased NPQ (as evidenced by the increase in the DES).

**Figure 3:**
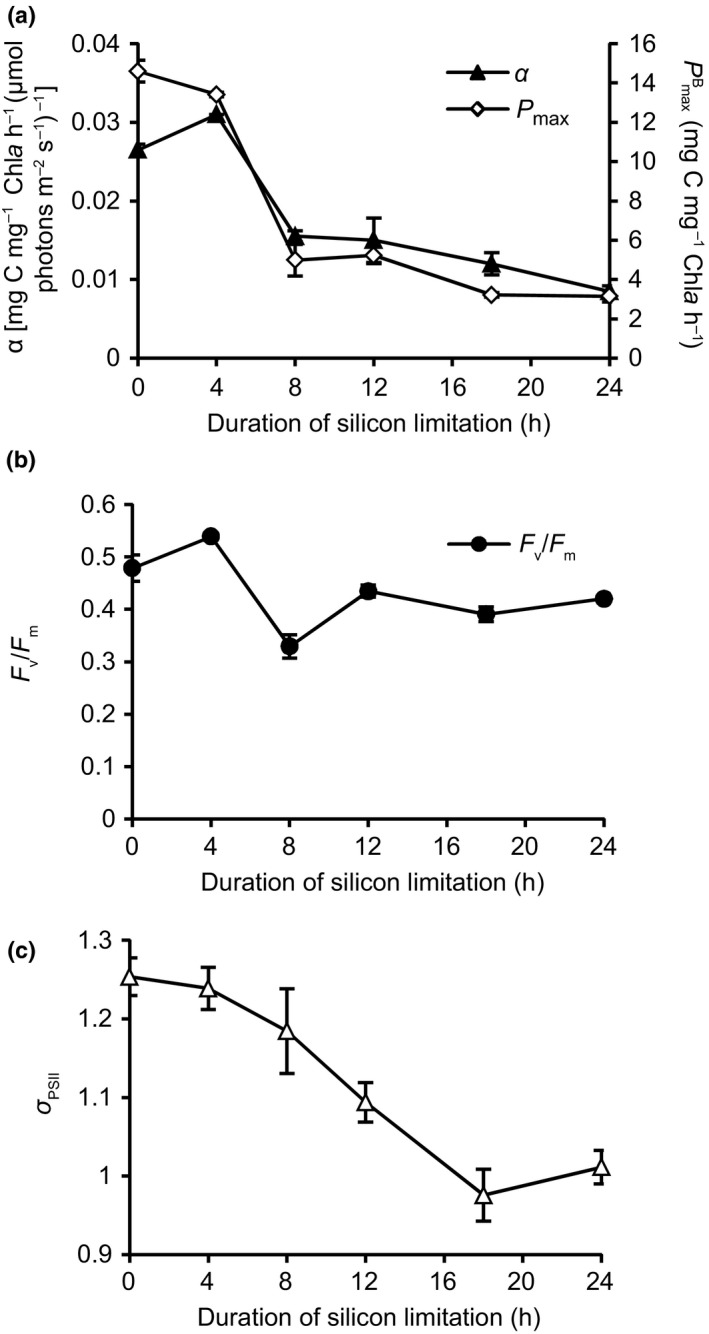
Photophysiological response of *Thalassiosira pseudonana* during silicon starvation. (a) *P*–*I* parameters *α* and $P_{\max}^{B}$ are shown. Data represent the average of two technical replicates from Si– #7. Error bars show ± SD. (b) *F*_v_/*F*_m_, representing the average of two technical replicates from Si– #7. (c) Absorption cross‐section (*σ* _PSII_); data represent the average of two technical replicates from Si– #7.

Several major shifts in photophysiological parameters were documented in response to silicon starvation‐induced growth arrest in experiment Si– #7. At 4 h of silicon starvation, the *P*–*I* parameter *α* increased relative to 0 h, indicating a transient increase in the efficiency of this transfer of energy (Fig. 3). At 8 h, both *α* and $P_{\max}^{B}$ declined, by 63% and 50%, respectively (Fig. 3a). Coincident with a decrease in *α* and $P_{\max}^{B}$, there was a significant decrease in *F*_v_/*F*_m_ at 8 h (Fig. 3b). This indicates that there was damage sustained at the photosystem II (PSII) reaction center core at 8 h, which at least partially affected carbon fixation at saturating light intensities at this time point. At 12 h, *F*_v_/*F*_m_ returned to *c*. 80% of initial values for the remainder of the experiment (Fig. 3b). This recovery of PSII is probably facilitated by an increase in NPQ as evidenced by the DES of the xanthophyll pool, which reduces the magnitude of the flow of damaging photons to PSII (Fig. 2b). Despite the recovery of *F*_v_/*F*_m_, *α* and $P_{\max}^{B}$ remained low for the remainder of the silicon starvation time‐course (Fig. 3a). The absorption cross‐section (*σ*_PSII_) decreased significantly during silicon starvation, indicating an overall reduction in light absorption (Fig. 3c).

**Figure 4:**
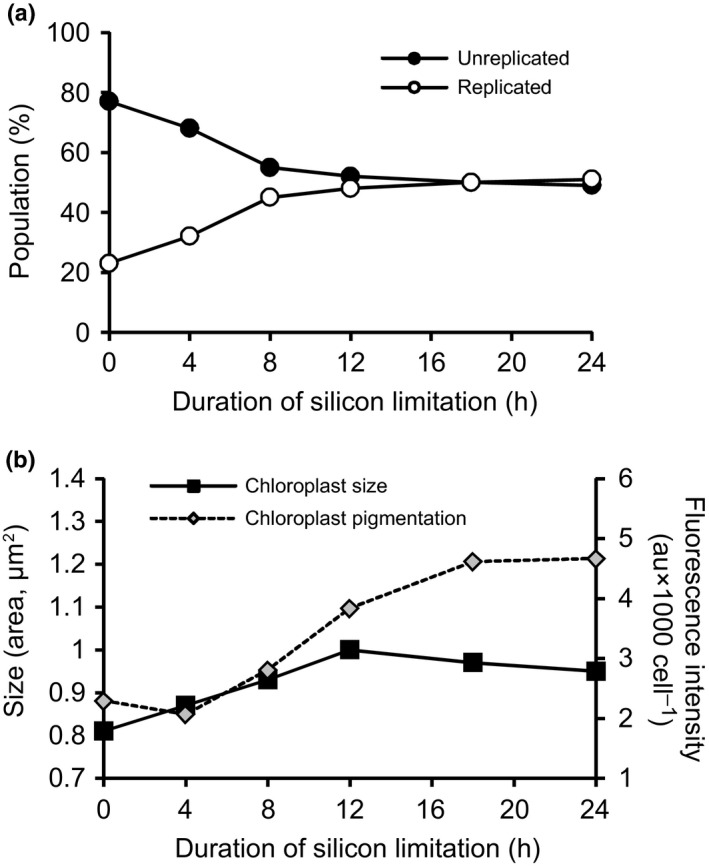
Imaging flow cytometric analysis of chloroplast number, size, and fluorescence intensity during silicon starvation. (a) Percentage of the *Thalassiosira pseudonana* population in possession of unreplicated (*n *=* *2) vs replicated (*n *=* *4) chloroplasts. Data represent values from an analysis of *n *>* *2000 images from Si– #8. (b) Average chloroplast size (area, μm^2^) is plotted along with chloroplast fluorescence intensity (in arbitrary units (au cell^−1^)). At each time point, data represent the average value from an analysis of *n *>* *2000 images from Si– #8.

Imaging flow cytometry was used to quantify the number, size, and fluorescence intensity of chloroplasts on the experimental time‐course to better interpret the observed pigment accumulation in silicon‐starved *T. pseudonana*. Initially, nearly 80% of the population possessed unreplicated chloroplasts; however, by 8 h, nearly half of the population had replicated chloroplasts (Fig. 4). Average chloroplast size increased between 0 and 12 h, and decreased at 18 and 24 h (Fig. 4b). At 8 h of silicon starvation, chloroplast fluorescence intensity increased for the duration of the experiment despite a decrease in the rate of division and size of chloroplasts, indicating that towards the later stages of silicon starvation, the organelles were becoming much more densely pigmented (Fig. 4b). Increased pigment content results from larger and replicating chloroplasts in the early stages of silicon starvation, and an overall increase in chloroplast pigmentation detected at 8–12 h.

### Genome‐wide and lipid‐specific transcript pattern analysis

The global transcriptomic response of silicon‐starved *T. pseudonana* was evaluated using both microarrays (Si– #3) and RNA‐Seq (Si– #9, Si– #10). Transcript abundance expression changes of 10 700 gene models from the Thaps3 and 245 gene models from the Thaps3_bd gene catalogs were compared (10 945 total). A large number of total gene models were significantly differentially expressed in both the microarray (*n *=* *5073) and RNA‐Seq (*n *=* *7024) experiments, and 4038 of those genes were common to both (Table S2). Transcript abundance patterns were grouped using *k*‐means clustering into 50 clusters (see the Materials and Methods; Table S2). Centroid value for each of the 50 clusters were used to calculate a PCC between the microarray and RNA‐Seq experiments. The majority of clusters were replicated (*n *=* *32, PCC > 0.5) between experiments. Both replicated and unreplicated clusters, which had high degrees of differential expression (log_2_ fold‐change > 1), and a high percentage of replicated genes within each cluster (> 50% of genes with PCC > 0.5, criteria for replicated clusters only) were included for further analysis (*n *=* *29; see the Materials and Methods).

**Figure 5:**
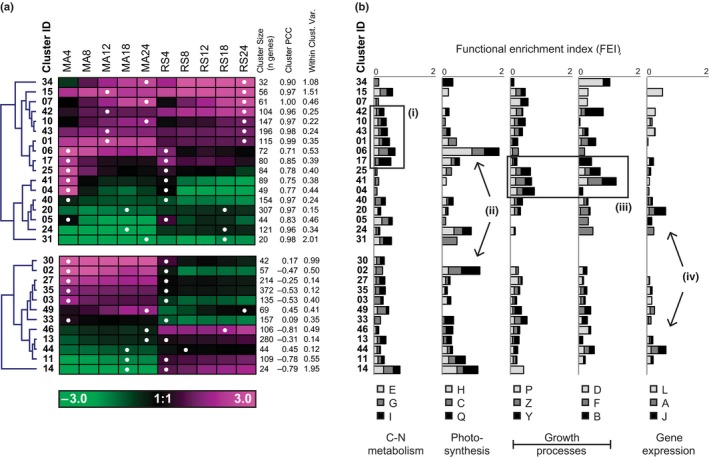
Gene expression clusters and functional annotation (KOG class) of genes clustered by expression pattern in *Thalassiosira pseudonana* during silicon starvation. (a) Heatmap for selected expression clusters representing the centroid log_2_ fold‐change in transcript abundance relative to 0 h at the five experimental time points for microarray data (MA4, MA8, MA12, MA18, MA24) and RNA‐Seq data (RS4, RS8, RS12, RS18, RS24). Numbers indicate hours of silicon starvation. Clusters were hierarchically clustered (shown in blue). The number of genes in each cluster, cluster Pearson correlation coefficient (between microarray and RNA‐Seq data), and within‐cluster variance are shown in the column to the right of (a). White dots indicate highest transcript abundance (relative to 0 h) for each cluster. Identity of genes within given clusters is shown in Supporting Information Table S2. Functional enrichment index for each expression cluster is plotted (b). KOG codes are used and are as follows: E, amino acid transport and metabolism; G, carbohydrate transport and metabolism; I, lipid transport and metabolism; H, coenzyme transport and metabolism; C, energy production and conversion; Q, secondary metabolite biosynthesis, transport and catabolism; P, inorganic ion transport and metabolism; Z, cytoskeleton; Y, nuclear structure; D, cell cycle control, cell division, chromosome partitioning; F, nucleotide transport and metabolism; B, chromatin structure and dynamics; L, replication, recombination and repair; A, RNA processing and modification; J, translation, ribosomal structure and biogenesis. Boxes (i, ii, iii, and iv) show regions of the plots referred to in the text. C–N, carbon–nitrogen.

The most strongly up‐regulated and replicated cluster of genes was cluster 15 (C15; Fig. 5a). Notably, C15 contained the silicon transporter SIT1 (Thaps3_268895) in addition to several other genes with roles in photorespiration and carbon concentration recently identified to be coexpressed by Hennon *et al*. (2015) (Fig. S2). Cluster 15 had high within‐cluster variance, so genes were regrouped (hierarchically) and subclusters were manually defined (Fig. S2). From this analysis, two genes were identified which were strongly coregulated with SIT1: one is a hypothetical protein (Thaps3_9619), and the other is a calcium‐dependent protein kinase (Thaps3_14322). The replicated and strong degree of coregulation of these transcripts is evidence that these genes are functionally related, and perhaps comprise a silicon sensing and/or response mechanism (Fig. S3).

Lipid metabolism genes were enriched in up‐regulated clusters, but were also found distributed across clusters with several different expression patterns (Fig. 5bi). Genes regulating the synthesis of polyunsaturated fatty acids (PUFAs) and other complex lipid biosynthesis (TAG, thylakoid lipids) were variably expressed during silicon starvation‐induced lipid accumulation; however, most of the transcript patterns were generally either not well correlated with observed biochemical shifts (i.e. PUFA concentrations) or were not well replicated between experiments (Fig. S4). Only a few genes coding components of complex lipid biosynthesis machinery were reproducibly replicated between experiments, including two TAG biosynthesis enzymes (MBOAT/DGAT1, Thaps3_261279, and LPLAT/AGPAT, Thaps3_261817, Fig. S4). These genes are the best candidates for having a role in the transcript level regulation of TAG synthesis during silicon starvation.

**Figure 6:**
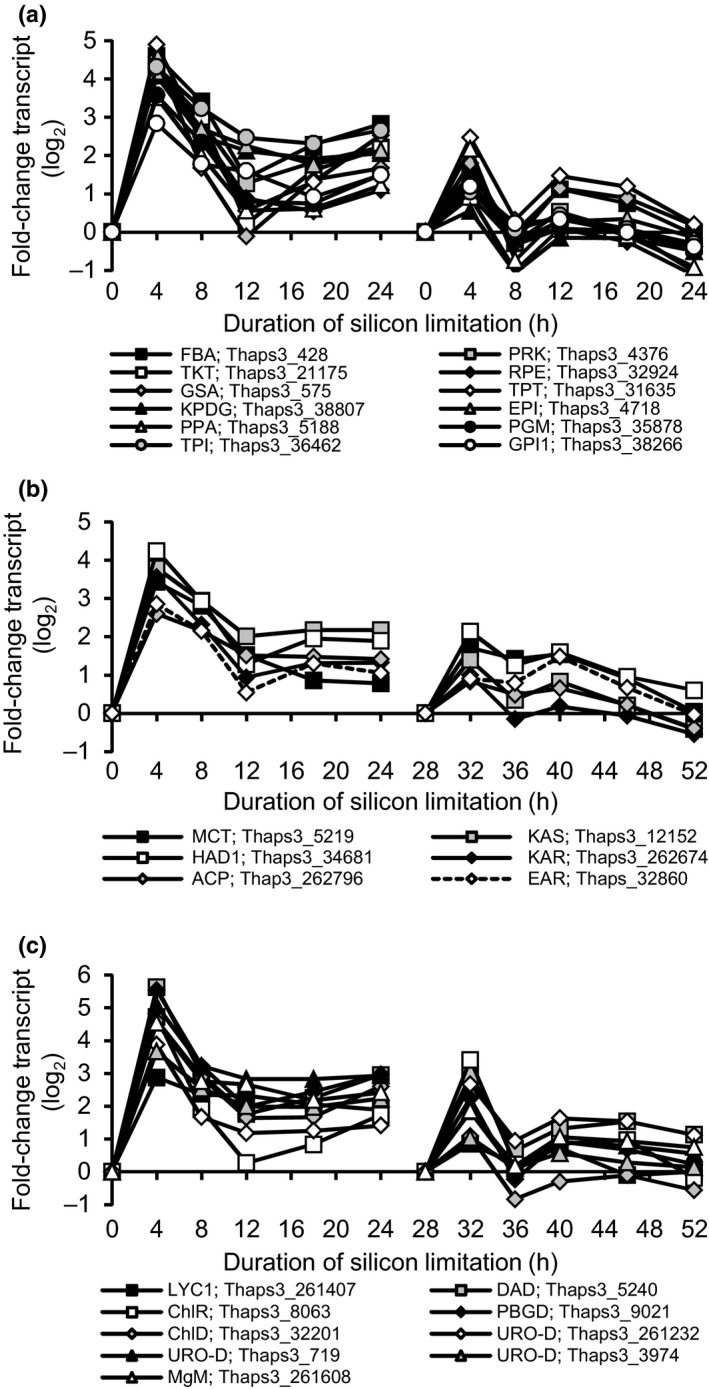
Coordinated changes in transcript abundance for cluster 6 genes coding key enzymes of carbon metabolism. (a–c) Transcript abundances of selected Calvin–Benson cycle and glycolysis enzymes (a), fatty acid biosynthesis enzymes (b), and pigment biosynthesis genes (c). Plots show both microarray data (left) and RNA‐Seq data (right). The dashed line in (b) indicates gene from cluster 17. Series labels show gene name and Thaps3 Protein ID (Table S2).

Several genes specifically involved in fatty acid biosynthesis and modification were differentially expressed (Fig. S4). *De novo* biosynthesis of fatty acids occurs in the chloroplast of diatoms via a type II fatty acid synthase (Armbrust *et al*., 2004) and the first committed step of fatty acid synthesis is catalyzed by acetyl‐coA carboxylase (ACCase). All components of this fatty acid synthesis machinery, including both chloroplast‐ and cytosol‐localized copies of ACCase (ACCase_chl_, Thaps3_6770 and ACCase_cyt_, Thaps3_12234, respectively), and nearly all of the enzymes catalyzing the subsequent steps of the fatty acid synthase pathway are up‐regulated (FASII; Fig. S4). Remarkably, nearly all FASII genes were found in a single coexpression cluster (C06; Fig. 6). This cluster was also highly enriched in genes for carbohydrate metabolism, coenzyme transport and metabolism, energy production and conversion, and secondary metabolite biosynthesis, transport, and catabolism (Fig. 5bii). These included key enzymes from diverse pathways including the Calvin–Benson cycle, glycolysis, and pigment biosynthesis, with nearly all the genes in this cluster predicted to be chloroplast‐localized (Fig. 6). In both microarray and RNA‐Seq experiments, genes in this cluster peak in transcript abundance at 4 h coincident with the timing of chloroplast replication (Fig. 4).

The 4 h differential expression maximum was a common feature characterizing the transcriptome of silicon‐starved *T. pseudonana* (Fig. 5a). Clusters with a 4 h peak were functionally enriched with genes for processes relating to the cytoskeleton, nuclear structure, nucleotide transport and metabolism, and chromatin structure and dynamics, including the core histones known to be tightly regulated at the transcript level to the early S‐phase of cell cycle progression (Fig. 5biii; Guglielmi *et al*., 2013). Translation, ribosomal structure, and biogenesis genes were down‐regulated, which is a characteristic feature of transcriptomes in cells with arrested cell growth and division (Fig. 5biv, Rudra & Warner, 2004). Together, these changes in transcript abundance are consistent with the timing of the observed partial cell cycle progression at 4 h and growth arrest (Fig. 1a,b).

### Evidence for a transcript level integrated high light stress response

**Figure 7:**
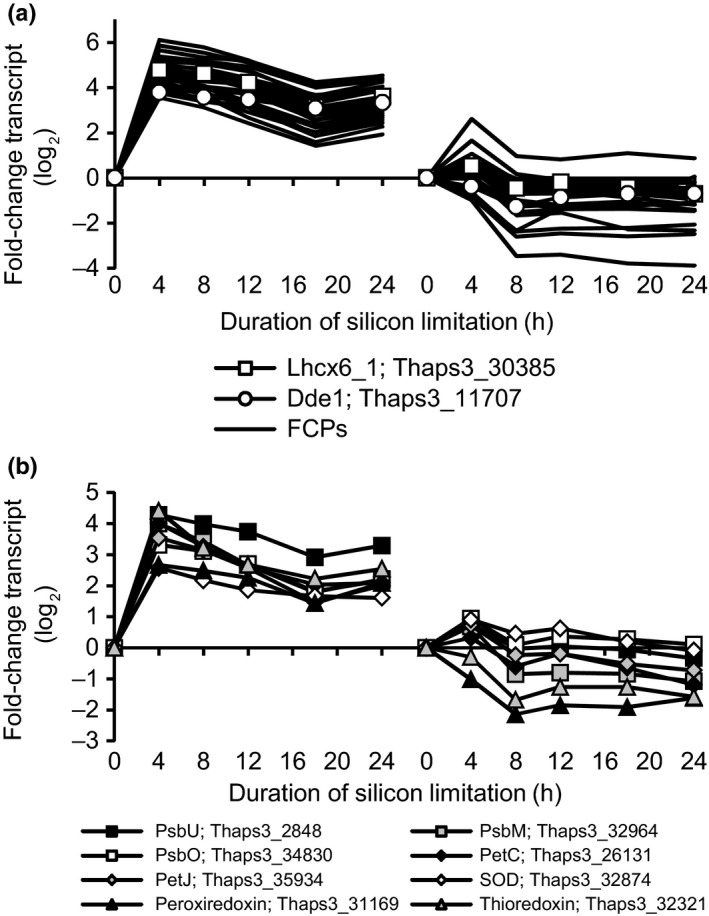
(a, b) Coordinated changes in transcript abundance for genes coding fucoxanthin‐chlorophyll proteins (FCPs) (a) and nuclear‐encoded components of the photosynthetic electron transport chain and redox‐sensitive genes (b). Plots show both microarray data (left) and RNA‐Seq data (right).

Although the genome‐wide transcriptomic response to silicon starvation and lipid accumulation was generally well replicated between microarray and RNA‐Seq experiments, there were several clusters in which changes in transcript abundance were not well replicated (Fig. 5a). Cluster 2 is an example of this, and is strongly enriched in genes involved in photosynthesis (Fig. 5bii). When combined with the nearly identically expressed cluster 30, these clusters comprised the majority (> 65%) of the annotated fucoxanthin Chl proteins (FCPs) in the *T. pseudonana* genome. This included *Lhcx6_1* (Thaps3_30385), an isoform of the Lhcx family of photoprotective proteins shown to be photoprotective and induced under high light stress in *T. pseudonana* (Zhu & Green, 2010)*,* and a putative diadinoxanthin de‐epoxidase, used to convert diadinoxanthin to diatoxanthin under high light (*Dde1*, Thaps3_11707; Fig. 7). These genes were much more strongly up‐regulated in the microarray experiment than in the RNA‐Seq experiments, indicating that this was not directly attributable to silicon starvation.

Changes in transcript abundance for genes for most of the other components of the photosynthetic electron transport (PET) chain (PSII, cytochrome *b*_6_f, PSI, ATP synthase) are encoded in the chloroplast genome of *T. pseudonana*, and therefore were only captured by the microarray. Generally, transcripts for PET components were up‐regulated, but not to the extent of the nuclear‐encoded FCPs (Table S2). Nuclear‐encoded constituents of the PET chain were up‐regulated in the microarray experiment similar to the pattern observed for the FCPs (Fig. 7a). The nuclear PET genes code for proteins likely to be utilized or damaged during periods of stress, such as PsbU, the 12 kDa extrinsic PSII protein required to stabilize the PSII oxygen‐evolving system in cyanobacteria (Nishiyama *et al*., 1997), and PsbO, an oxygen‐evolving enhancer protein. Superoxide dismutase (Thaps3_32874) and redox‐signaling proteins like peroxiredoxin (Thaps3_31169) and thioredoxin (Thaps3_32321) were also coordinately up‐regulated with other putative high light responding genes, consistent with an increased need to cope with and react to an enhanced flux of electrons through the PET chain during these conditions.

**Figure 8:**
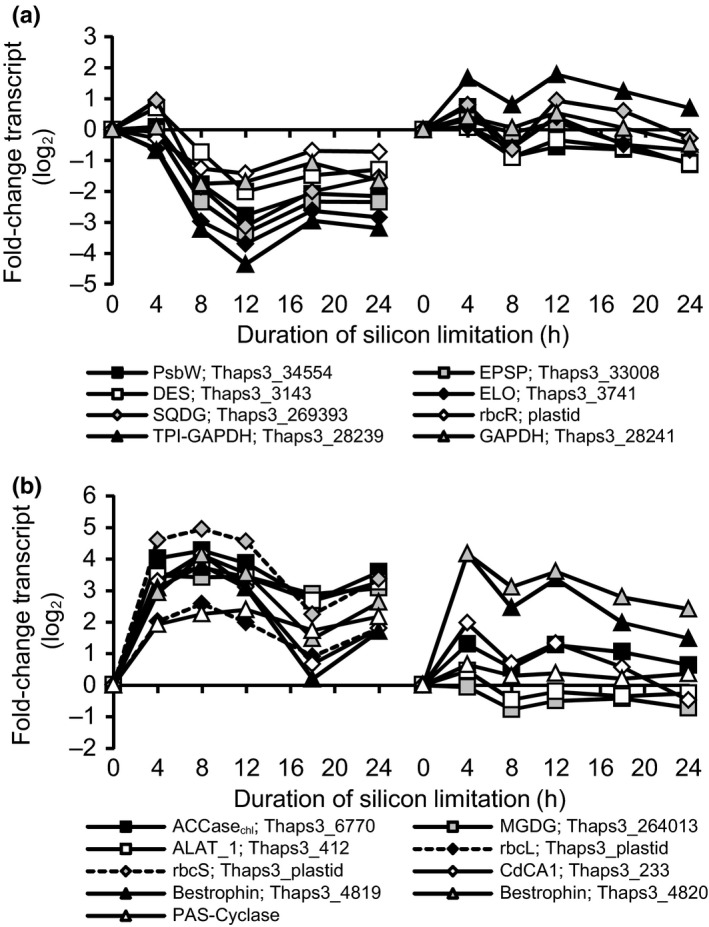
(a, b) Coordinated changes in transcript abundance of carbon metabolism genes coexpressed with ACCase_chl_ (a) and down‐regulated genes involved in various aspects of carbon and nitrogen metabolism (b). Plots show both microarray data (left) and RNA‐Seq data (right). Series labels show gene name and Thaps3 Protein ID (Table S2). Dashed lines indicate that microarray values for rbcL and rbcS are transformed (×10) to fit on the same axes.

Interestingly, the only down‐regulated components of the PET chain in the microarray experiment were both the nuclear and plastid copies of PsbW (Thaps3_34554, plastid_psbW, Cluster 5, Fig. 8a). This gene is in the process of being transferred from the chloroplast to the nuclear genome, and though the function of this protein is unknown, it is thought to stabilize the PSII protein complex (Armbrust *et al*., 2004; García‐Cerdán *et al*., 2011). The decoupling of its expression from all other components of PSII in the microarray experiment suggests it may have a unique role in light harvesting or photoprotection.

### Coordinated regulation across energy and carbon pathways during metabolic shifts

Silicon starvation induces several metabolic shifts in *T. pseudonana* across several aspects of cellular growth and metabolism, and the way in which the shifts in these processes are coordinated is largely unknown. Some gene clusters were clearly enriched in genes with a particular biological function (i.e. photosynthesis, light harvesting), but there were several clusters of replicated genes that did not appear to be enriched in any particular biological function (Fig. 5). More detailed analysis revealed that genes often attributed to various cellular energy and carbon sources and sinks were found within given clusters, suggesting that there is a transcript level integration of the regulation of these processes.

Cluster 5 (*n *=* *44 genes) contains several genes involved in processes ranging from photosynthesis to cell cycle progression (Fig. 5). Genes in this cluster include PsbW (Thaps3_34554), a key enzyme in aromatic amino acid biosynthesis EPSP synthase (Thaps3_33008), and several genes with key roles in the synthesis of complex lipids, including a fatty acid desaturase (Thaps3_3143), elongase (Thaps3_3741), and sulfoquinovosyl diacylglycerol (SQDG) synthase (Thaps3_269393), which synthesizes a thylakoid sulfolipid that inhibits diadinoxanthin de‐epoxidation (Goss *et al*., 2009; Fig. 8a). The plastid‐encoded transcriptional regulator of the RuBisCO operon (RbcR) is also similarly down‐regulated. Transcript abundances of the mitochondrial isoforms of glyceraldehyde 3‐phosphate dehydrogenase (GAPDH, Thaps3_28241, C37) and a triosephosphate isomerase‐GAPDH fusion protein (TPI‐GAPDH, C14, Thaps3_28239) were coordinated with the transcript abundance changes observed in cluster 5, more strongly in the microarray experiment than in the RNA‐Seq experiment. Owing to their mitochondrial localization and conservation throughout diatom evolution, these isoforms of key glycolysis enzymes are thought to be disproportionately important in the supply of carbon to the  tricarboxylic acid (TCA) cycle for cellular respiration, thereby providing both energy and molecular precursors necessary for growth and division (Smith *et al*., 2012). Reasons underlying the stronger down‐regulation of cluster 5 in the microarray experiment are unknown, but regardless of this difference, it is clear that in both experiments there is coordination of these genes at the transcript level. Considering their biological roles, it is possible that this coregulation occurs to integrate several processes required for normal cell proliferation.

Changes in transcript abundance of ACCase_chl_ (Thaps3_6770), the first committed step of chloroplast fatty acid biosynthesis, are strikingly correlated with changes in transcript abundance of several other genes, including monogalactosyldiacylglycerol (MGDG) synthase (Thaps3_264013), which synthesizes the thylakoid neutral galactolipid MGDG, and a putative cytosolic alanine aminotransferase (ALAT_1; Thaps3_412, Fig. 8b). Additionally, ACCase_chl_ is coordinately expressed with both of the plastid‐encoded subunits of RuBisCO (rbcS, rbcL). RuBisCO and ACCase_chl_ are carboxylases requiring inorganic carbon (CO_2_ or bicarbonate) as a substrate, suggesting that inorganic carbon availability may be a factor regulating the transcript abundance of these genes.

Recently, a CO_2_‐responsive coexpression cluster of several genes involved in carbon‐concentrating mechanisms/photorespiration was documented (Hennon *et al*., 2015). Although the complete cluster observed in Hennon *et al*. (2015) was not recovered as a single cluster in our analysis, several of these genes were coexpressed in silicon‐starved *T. pseudonana,* including an isoform of δ‐carbonic anhydrase (Thaps3_233), and two bestrophins (Thaps3_4819, Thaps3_4820), which are predicted to be chloroplast‐localized, up‐regulated at the protein and transcript levels under low CO_2_ conditions, and hypothesized to play a role chloroplast inorganic carbon supply (Kustka *et al*., 2014; Hennon *et al*., 2015). The promoters of these genes are reported to contain potential *cis‐*regulatory motifs which confer sensitivity to CO_2_‐induced shifts in intracellular cAMP concentrations (CCREs; Ohno *et al*., 2012; Hennon *et al*., 2015). Although these genes did not cluster with ACCase_chl_, they were expressed with a very similar pattern (Fig. 8b). The upstream regions (800 bp) of ACCase_chl_, MGDG synthase (Thaps3_264013), and ALAT_1, were examined for the presence of CCREs, to explore the possibility that transcription of genes is mediated by the same transcription factor as the genes identified by Hennon *et al*. (2015). The ACCase_chl_ promoter had both CCRE1 and CCRE2 elements +248 nt upstream of the predicted open reading frame, but neither MGDG synthase (Thaps3_264013) nor ALAT_1 promoters had CCREs, meaning that a distinct mechanism (i.e. transcription factor) may function to coordinate the transcription of these genes with the CO_2_ cluster.

Genes with CCREs are transcriptionally repressed by cAMP, suggesting that during silicon starvation, there is a decrease in cAMP concentrations between 4 and 12 h in the microarray experiment, and fluctuating cAMP concentrations between 4 and 12 h in the RNA‐Seq experiments. The specific cyclases and phosphodiesterases responsible for regulating intracellular cAMP concentrations in diatoms are unknown, although candidate CO_2_‐sensing cyclases have been proposed (Hennon *et al*., 2015). A putative adenylate/guanylate cyclase was identified in this study (Thaps3_262719), and was coexpressed with the CO_2_‐sensitive/ACCase_chl_ cluster (Fig. 8). This gene model was incomplete, but an alternative gene model (Thaps3_5951) combines Thaps3_262719 with two upstream gene models (Thaps3_262720, Thaps3_262721), both of which contain PAS domains that function as sensors and are involved in cell signaling. This single gene model (PAS‐cyclase) is supported by our RNA‐Seq data, and predicted to be cytosolic as no subcellular localization could be predicted bioinformatically (Fig. S5). The coexpression of this PAS‐cyclase with inorganic carbon fixation genes, photorespiration genes, and carbon concentrating mechanism genes is good evidence supporting a role in mediating signal transduction within the cell during low‐CO_2_ conditions. The promoter region of PAS‐cyclase does not contain any CCREs, suggesting its own transcription is decoupled directly from CCRE‐mediated cAMP regulation.

## Discussion

### Cell cycle as a regulator of transcript abundance and lipid content

Nutrient stress in diatoms induces changes in transcript abundances and can cause dramatic shifts in cellular health and physiology (Allen *et al*., 2008; Mock *et al*., 2008; Dyhrman *et al*., 2012; Hockin *et al*., 2012; Bender *et al*., 2014; Abida *et al*., 2015). Studies aimed at elucidating changes in transcript abundance during nutrient‐limited lipid accumulation often use nitrogen as the limiting nutrient, which is a stressful condition and induces massive cellular changes such as chlorosis and an associated down‐regulation of photosynthesis (Sun *et al*., 2013; Abida *et al*., 2015; Levitan *et al*., 2015). In contrast, short‐term (24 h) silicon starvation has been used as a method to synchronize diatom cultures, and is not generally considered a stressful condition (Coombs *et al*., 1967; Hildebrand *et al*., 2007; Shrestha *et al*., 2012). Silicon starvation‐induced changes in transcript abundance are more likely to reflect the cellular processes that occur as *T. pseudonana* arrests growth, rather than a response to stress.

**Figure 9:**
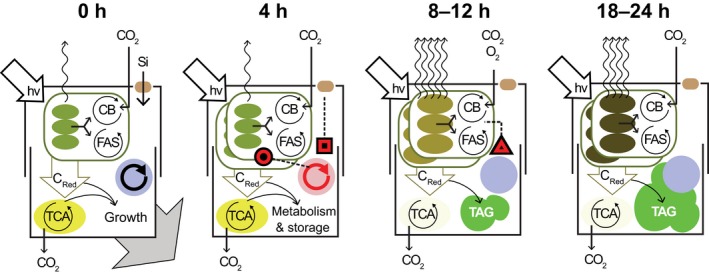
Conceptual overview summarizing major changes in carbon and energy flux, and putative regulatory events in silicon‐starved *Thalassiosira pseudonana* (experiments Si– #1–8) at 0, 4, 8–12, and 18–24 h. At 0 h, light absorption, carbon fixation, and respiration are in balance for growth, and silicon transporters sense silicic acid. Transcription in the nucleus (blue) is regulated by cell cycle progression, and light energy lost via nonphotochemical quench (NPQ, wavy arrow) is low. At 4 h, cells sense reduced silicon availability and signal the nucleus through a putative silicon detection mechanism (black and red box; Fig. S3). Growth is arrested, but cell cycle proceeds partially and signals chloroplast replication (black and red circle). At 8–12 h, pigment composition has shifted towards an increased proportion of photoprotective pigments, and the de‐epoxidation state (DES) has increased, indicating an increase in loss of light energy through NPQ. A decrease in cellular cAMP concentrations (mediated by low CO_2_) signals increased transcription of photorespiration/carbon concentrating/ACCase_chl_ (black and red triangle). Flux of carbon towards the tricarboxylic acid (TCA) cycle is down‐regulated and triacylglycerol (TAG) droplets form. By 18–24 h, chloroplasts have become more heavily pigmented and have remodeled to increase NPQ, cell cycle is completely arrested, and cells continue to accumulate TAG droplets. CB, Calvin–Benson; FAS, fatty acid synthesis; C_Red_, reduced carbon.

How diatoms sense and respond to nutrient deprivation is largely unknown. Recent work by Shrestha *et al*. (2015) show that knockdown of silicic acid transporters (SITs) results in an earlier onset in lipid accumulation under silicon starvation than wild‐type *T. pseudonana,* indicating the presence of a silicon‐sensing mechanism. We identified genes that are highly up‐regulated and coexpressed with SIT1 during silicon starvation, including a protein kinase (Thaps3_14322) and a hypothetical protein (Thaps3_9619). Transcripts of these genes are down‐regulated during silicon recovery (Shrestha *et al*., 2012), and are reproducibly coexpressed (Hennon *et al*., 2015). We hypothesize that these genes are part of a silicon‐sensing and response mechanism, and, through this sensor activity, could initiate a transcriptional cascade before the onset of actual silicon limitation (Fig. 9).

Cellular growth and chloroplast replication are tightly regulated cellular processes (Wittenberg & Reed, 2005; Hudik *et al*., 2014) and are shown to be coordinated in diatoms (Gillard *et al*., 2008). A strong transcript level response at 4 h of silicon starvation was documented in *T. pseudonana* and occurred while nearly half of the population continued to progress through the cell cycle (Fig. 1b). Genes with this expression pattern were functionally enriched for cell cycle progression‐related phenomena, strongly supporting a role for transcript level regulation in coordinating associated processes. Many of these genes were also part of chloroplast‐localized pathways. The high degree of correlation of transcript abundances with chloroplast replication but not with corresponding physiological changes (carbon fixation rates, fatty acid shifts, and pigments) supports the possibility that transcripts are up‐regulated to enzymatically populate dividing chloroplasts. We conclude that regulation of the transcript abundance of genes with this characteristic response is accomplished by mechanisms in place to regulate and coordinate cell cycle progression/chloroplast replication, rather than by factors such as reduced silicon availability, stress, or a strategy to regulate the observed shifts in carbon metabolism.

Despite the high reproducibility of the lipid accumulation response, transcript level changes of genes involved in complex lipid metabolism were generally poorly replicated between experiments, suggesting an unimportant role of transcript level regulation in controlling triacylglycerol (TAG) biosynthesis, with the exception of MBOAT/DGAT1 (Thaps3_261279) and LPLAT/AGPAT (Thaps3_261817). Previous studies in microalgae have similarly found that changes in transcript abundance for lipid metabolism genes do not necessarily directly reflect lipid induction state, suggesting that expression of these enzymes may not regulate flux of intracellular carbon into TAG (Radakovits *et al*., 2012; Liang *et al*., 2013; Li *et al*., 2014; Tanaka *et al*., 2015). However, several recent genetic manipulations of a number of *T. pseudonana* lipid metabolism enzymes with variable or unreplicated transcript level responses in this experiment have been shown to affect lipid content and composition (Trentacoste *et al*., 2013; Cook & Hildebrand, 2015; Manandhar‐Shrestha & Hildebrand, 2015). A better understanding of the biochemistry and regulation of lipid metabolism in algae is needed to effectively interpret the significance of transcript level data and predict outcomes of genetic manipulations (Rismani‐Yazdi *et al*., 2011; Rizmani‐Yazdi *et al*., 2012; Lv *et al*., 2013; Gao *et al*., 2014; Li *et al*., 2014).

Lipid synthesis is regulated alongside cell cycle in order to satisfy cellular demand for phospholipids during growth and membrane biosynthesis (Jackowski, 1996). We suggest that the observed TAG accumulation after 8–12 h of starvation in *T. pseudonana* is the result of ongoing production of fatty acids with a decrease in cellular requirements for membrane biosynthesis. Other studies have shown that after returning to nutrient‐replete conditions after a short exposure to depleted conditions, diatoms rapidly draw down accumulated lipid stores and resume growth (Coombs *et al*., 1967; Valenzuela *et al*., 2013). Bioenergetically, it would be more efficient for these stored lipids to be utilized as precursors for membranes or other molecules rather than to be catabolized completely. Only the earliest stages of lipid accumulation in *T. pseudonana* were investigated. It is possible that different nutrient limitations on varying timescales can regulate the size of the cellular TAG pool through other mechanisms (Traller & Hildebrand, 2013). In addition to serving as a reservoir for carbon following catabolism or as a supply of macromolecular precursors, carbon stored in TAG droplets can also be used as an energy source for algae during long‐term survival of unfavorable conditions (Wang *et al*., 2009; Liu & Benning, 2013). Ultimately, it is likely that there are diverse cellular mechanisms that govern the storage of cellular carbon as TAG, which is an important consideration when selecting targets to engineer algae for enhanced lipid productivity (Cooksey, 2015).

### Photophysiological shifts during silicon starvation

Increases in cellular pigmentation and chloroplast replication have previously been documented under silicon‐induced growth arrest in diatoms (Holmes, 1966; Coombs *et al*., 1967; Harrison *et al*., 1977; Traller & Hildebrand, 2013). This is in strong contrast to the response of nitrogen limitation in which many photosynthetic taxa become chlorotic (Abida *et al*., 2015). Here, we show chloroplasts also became more heavily pigmented, the absorption cross‐section (*σ*_PSII_) decreased, and pigment composition shifted towards a higher proportion of xanthophyll pigments. In the microarray experiment, transcript abundances of the majority of outer antenna constituents also increased, although a similar up‐regulation was not observed in the RNA‐Seq experiment, indicating that this response is not necessarily related to silicon starvation or lipid accumulation. In both experiments, cells were harvested at a concentration of *c*. 1 × 10^6^ cells ml^−1^ and resuspended at 5 × 10^5^ cells ml^−1^ (Si– #1–8) and 1 × 10^6^ cells ml^−1^ (Si– #9–10). We attribute the variability in the expression of the outer antenna to a difference in self‐shading, owing to differences in experimental cell concentration. In the microarray experiment (Si– #3), the cells would experience a higher light stress, relative to their adapted state, than cells from the RNA‐Seq experiments (Si– #9–10).

Hallmarks of high light stress were observed in silicon‐starved *T. pseudonana*, such as increased NPQ, and transcript level induction of *Lhcx6_1*. Paradoxically, under these conditions, *T. pseudonana* increased cellular pigmentation and up‐regulated transcripts for the outer light‐harvesting antenna. Increases in cellular pigmentation are typically observed during low light adaptation to increase the absorption cross‐section (*σ*_PSII_) and maximize the flux of photons to photosynthetic electron transport (Falkowski & Raven, 2007). Arresting growth of *T. pseudonana* with silicon starvation disrupts balance between energetic and carbon inputs/outputs (i.e. NPQ, growth, respiration, metabolite exudation) established during exponential growth (Fig. 9). *T. pseudonana* must both redistribute these resources into carbon storage and reduce energy inputs through increasing NPQ (Fig. 9). We suggest that this energetic rebalancing is accomplished in part by an increase in pigmentation to protect the photosynthetic machinery by self‐shading (i.e. the package effect; Dubinsky *et al*., 1986; Falkowski & LaRoche, 1991). Furthermore, by increasing xanthophyll pigment concentrations in the total pigment pool, excess light energy that is harvested by the more heavily pigmented antenna could be funneled to antenna‐quenching sites rich in xanthophyll pigments rather than to PSII, restoring the balance of electron supply to PSII with PET (Lepetit *et al*., 2012; Goss & Lepetit, 2015).

Signals from the chloroplast (metabolite concentrations, redox state, etc.) communicate with the nucleus to regulate nuclear transcription and integrate chloroplast and nonchloroplast metabolism (Rüdiger & Oster, 2012; Rochaix & Ramundo, 2014). Reduced PET as a result of damage to PSII detected at 8 h of silicon starvation could result in an overall shift in the redox state of the chloroplast (Pfalz *et al*., 2012). *P*‐*I* parameters *α* and *P*_max_ also decreased at 8 h, from a limit in the supply of ATP and NADPH from impaired PET, a reduction in the supply of intermediates required for carbon fixation, and/or an increase in oxygen fixation by RuBisCO. Changes in chloroplast physiology and metabolism, such as a decrease in redox state from PSII damage, or any of the factors that impact *P*‐*I* parameters, at around the 8 h time point could serve as important signals that trigger specific signal transduction mechanisms to coordinate and regulate other nonchloroplast processes in the second half of the silicon starvation time‐course in *T. pseudonana*.

### The significance of highly coordinated gene expression clusters

During silicon starvation and lipid accumulation, the most conspicuous feature of the *T. pseudonana* transcriptome was the degree to which large clusters of genes were coordinately regulated. These suites of metabolically and functionally diverse genes are coordinated by unknown master regulators that could be as specific as transcription factors but are more likely to be broad cellular signals such as redox state, intracellular metabolite concentrations, light, cell cycle, or cAMP concentrations (regulated by proteins such as the described PAS‐cyclase). Organizing genes into large coexpression clusters that can be driven by these broad signals gives eukaryotic organisms the ability to coarsely regulate and orchestrate cellular processes such as growth or environmental adaptation. Finer‐scale transcriptional regulation (i.e. at the level of the transcription factor) operates within this large‐scale gene regulatory framework.

With the increasing availability of time‐course‐resolved transcriptomes, it is becoming more apparent that different organisms follow highly choreographed transcriptional programs by which large suites of genes are coordinated (Zinser *et al*., 2009; Monnier *et al*., 2010; Singh *et al*., 2010; Ashworth *et al*., 2013; Chauton *et al*., 2012; Dyhrman *et al*., 2012; Kanesaki *et al*., 2012; Nymark *et al*., 2013; Hennon *et al*., 2015; Poliner *et al*., 2015). In these cases, transcript level fluctuations are often correlated with light. Light is a powerful driver of genome‐wide transcriptome fluctuations both directly (i.e. through light sensors such as AUREOCHROME) and indirectly through metabolic shifts, internal pH shifts, and redox. In diatoms, the most well resolved regulators of cell cycle progression are light‐sensitive proteins (Huysman *et al*., 2013, 2014). In this study, there are similarly choreographed changes to the *T. pseudonana* transcriptome, but under continuous light conditions, illustrating the existence of other regulators that control coexpression modules besides light fluctuations.

Insights into the architecture of gene regulation at the transcript level can be gained by the identification of coexpression clusters. In this experiment, certain genes are clearly coexpressed with one another at the transcript level. Coregulation of genes at the transcript level supports their functional association and provides an insight into their biological roles, even without knowing the significance of up‐ or down‐regulation of transcripts, or the identity of the master regulator that integrates this expression. Careful dissection of the gene regulatory architecture in diatoms will ultimately require studies utilizing new and evolving methods aimed at investigating specific features of gene expression. Time‐course transcriptomes are useful to reveal features of the organization of gene regulatory networks in response to environmental perturbations, such as those introduced through silicon starvation‐induced growth arrest.

## Author contributions

S.R.S., C.G. M.H., R.M.A. and J.C.T. planned, designed, and performed the research. A.E.A. designed the microarrays, produced the microarray data, and performed bioinformatics analysis for the microarray data. R.M.A. performed bioinformatics analysis for the RNA‐Seq data. S.R.S., C.G., R.M.A., J.C.T., A.D., E.T., M.H. and M.V. analyzed the data. S.R.S., C.G., R.M.A., J.C.T., A.D., M.H., M.V. and A.E.A. wrote the paper.

## Supporting information

Please note: Wiley Blackwell are not responsible for the content or functionality of any supporting information supplied by the authors. Any queries (other than missing material) should be directed to the *New Phytologist* Central Office.

**Fig. S1** Comparison of microarray and RNA‐Seq data.
**Fig. S2** Cluster 15 gene expression subclusters.
**Fig. S3** Coordinate regulation of a putative silicon sensing mechanism.
**Fig. S4** Lipid metabolism gene expression.
**Fig. S5** PAS‐cyclase gene model.
**Table S1** Summary of silicon starvation experiments conducted.
**Methods S1** Detailed methods.

**Table S2** Microarray and RNA‐Seq data.
