# Supplementary material for: Transcript level coordination of carbon pathways during silicon starvation‐induced lipid accumulation in the diatom Thalassiosira pseudonana
Source: New Phytol. 2016 Feb 4;210(3):890–904. doi: 10.1111/nph.13843 (PMC5067629; doi:10.1111/nph.13843)
Supplement: Supplementary file 1 — Fig. S1 Comparison of microarray and RNA‐Seq data. Fig. S2 Cluster 15 gene expression subclusters. Fig. S3 Coordinate regulation of a putative silicon sensing mechanism. Fig. S4 Lipid metabolism gene expression. Fig. S5 PAS‐cyclase gene model. Table S1 Summary of silicon starvation experiments conducted. Methods S1 Detailed methods. [file NPH-210-890-s001.pdf]

New Phytologist Supporting Information Figs S1–S5, Tables S1 & S2 and Methods S1

Article title: Transcript level coordination of carbon pathways during silicon starvation induced lipid accumulation in the diatom *Thalassiosira pseudonana*

Authors: Sarah R. Smith, Corine Glé, Raffaela M. Abbriano, Jesse C. Traller, Aubrey Davis, Marina Vernet, Emily Trentacoste, Andrew E. Allen and Mark Hildebrand

Article acceptance date: 3 December 2015

The following Supporting Information is available for this article:

Fig. S1 Comparison of microarray and RNA-Seq data.

Fig. S2 Cluster 15 gene expression subclusters.

Fig. S3 Coordinate regulation of a putative silicon sensing mechanism.

Fig. S4 Lipid metabolism gene expression.

Fig. S5 PAS cyclase gene model.

Table S1 Summary of silicon starvation experiments conducted

Table S2 Microarray and RNA-Seq data (separate Excel file)

Methods S1 Detailed methods.

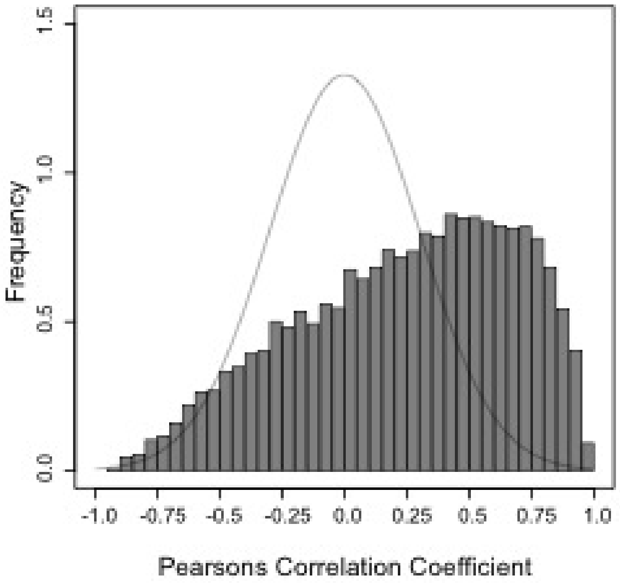

Fig. S1 Comparison of microarray and RNA-Seq data. Data show frequency distribution of Pearson correlation coefficients calculated using core signal fluorescence intensities (from the microarray) and the DESeq2 normalized counts (from RNA-Seq) for 10,945 gene models.

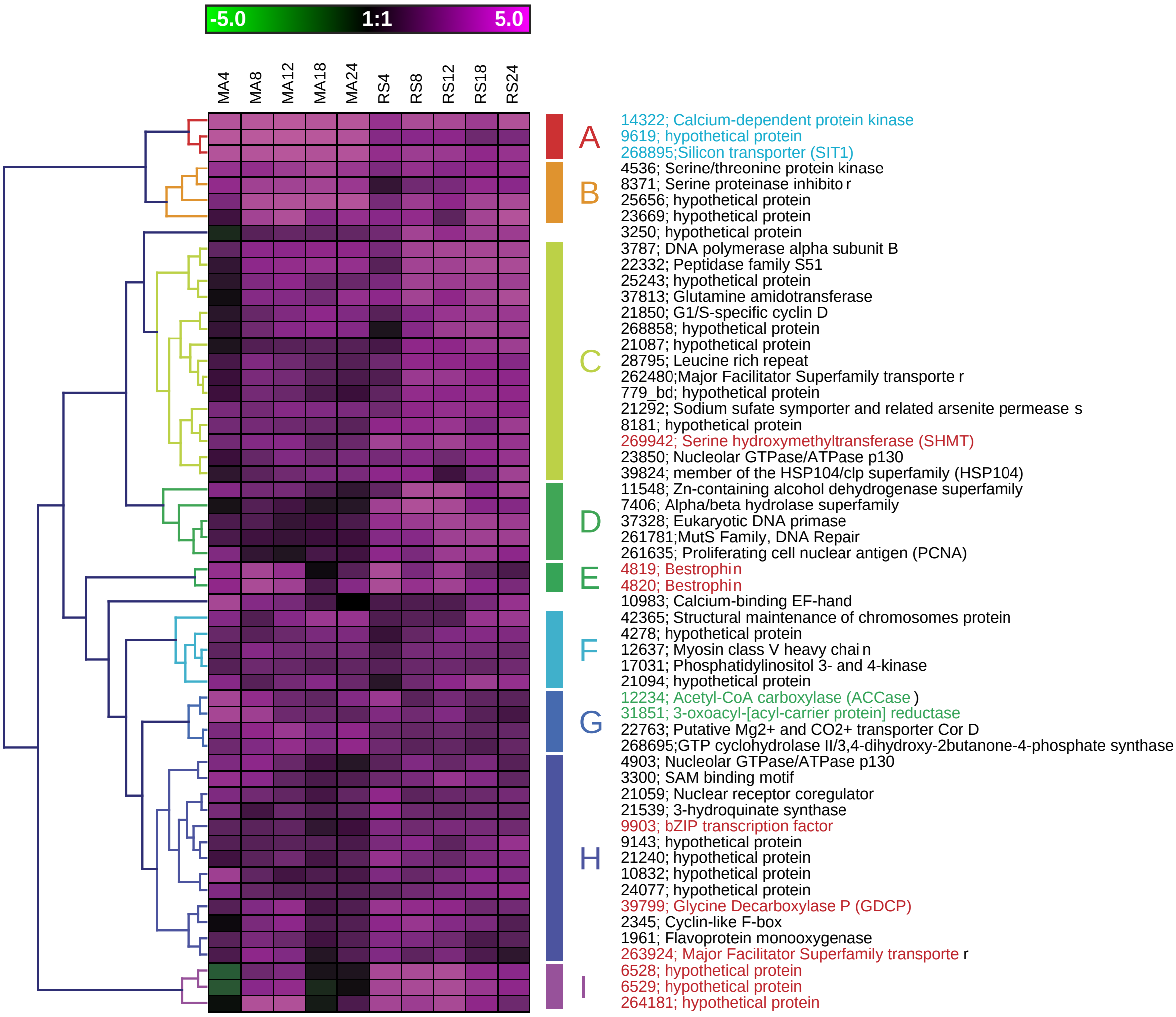

Fig. S2 Cluster 15 gene expression sub-clusters. Heatmap of log2 fold-change transcript data for cluster 15. Columns indicate experimental time points for microarray data (MA4, MA8, MA12, MA18, MA24) and RNA-Seq data (RS4, RS8, RS12, RS18, RS24). Numbers indicate hours of silicon starvation. Genes were hierarchically clustered and expression cluster cut-offs set manually. Labels show Thaps3 protein IDs and putative gene identities. Genes shown in red were coexpressed in Hennon *et al.* (2015). Genes shown in blue are a putative silicon sensing mechanism (Fig. S3). Genes shown in green are the cytosolic isoform of ACCase and a coexpressed chloroplast-localized keto-acyl-reductase of fatty acid biosynthesis.

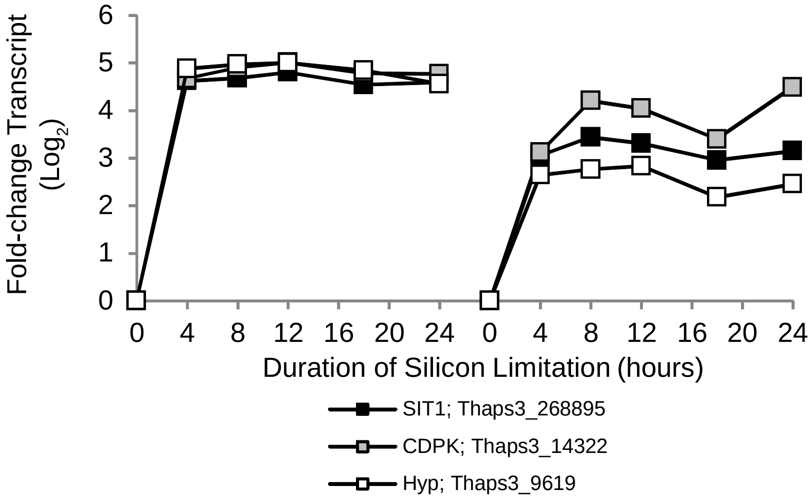

Fig. S3 Coordinate regulation of a putative silicon sensing mechanism. Plot shows both microarray data (left) and RNA-Seq data (right). Series labels show gene name and Thaps3 protein ID (Table S2).

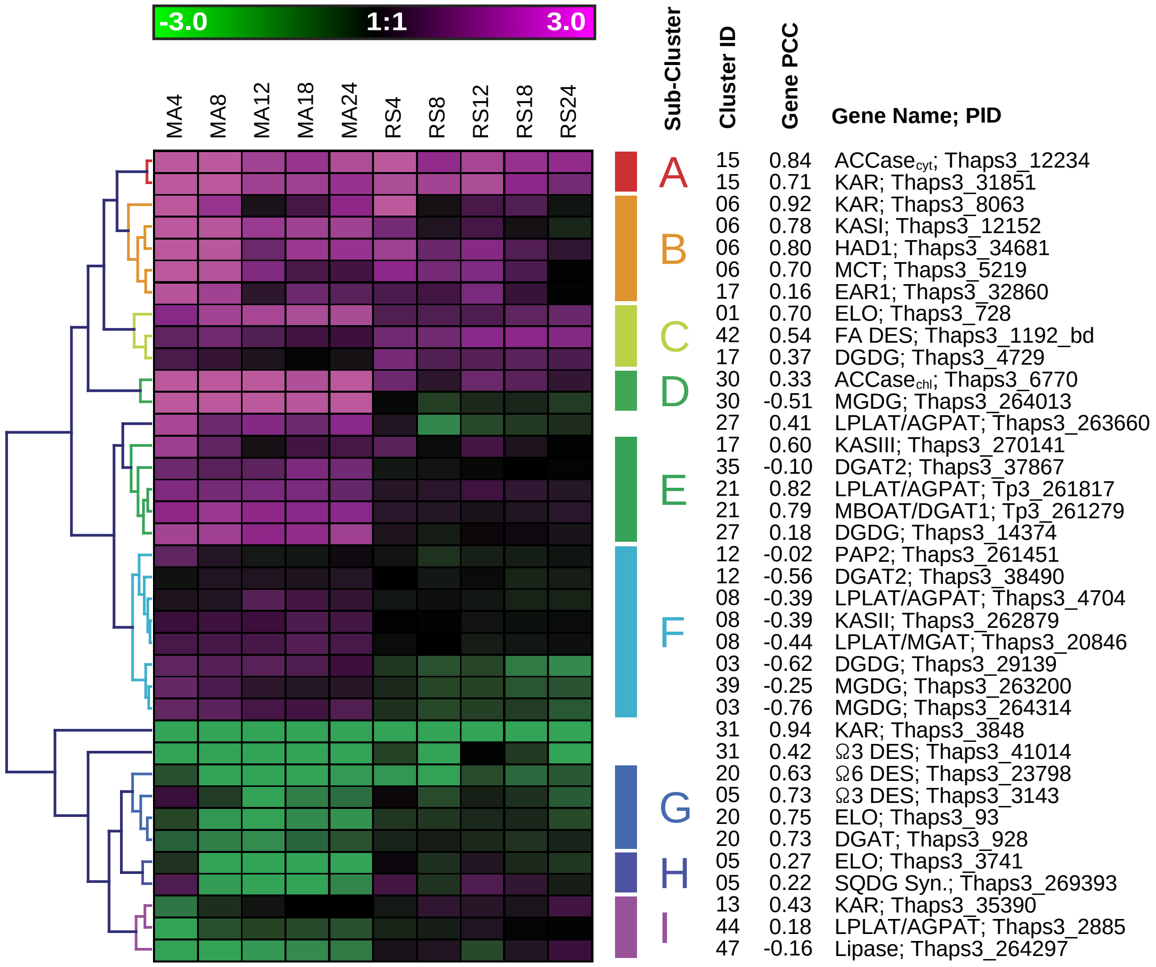

Fig. S4 Lipid metabolism gene expression. Heatmap of log2 fold-change transcript data for lipid metabolism genes. Columns indicate experimental time points for microarray data (MA4, MA8, MA12, MA18, MA24) and RNA-Seq data (RS4, RS8, RS12, RS18, RS24). Numbers indicate hours of silicon starvation. Genes were hierarchically clustered and expression cluster cut-offs set manually. Clusters, and pearson correlation coefficients between microarray and RNA-Seq experiments are indicated. Labels show gene names and Thaps3 protein IDs.

(a)

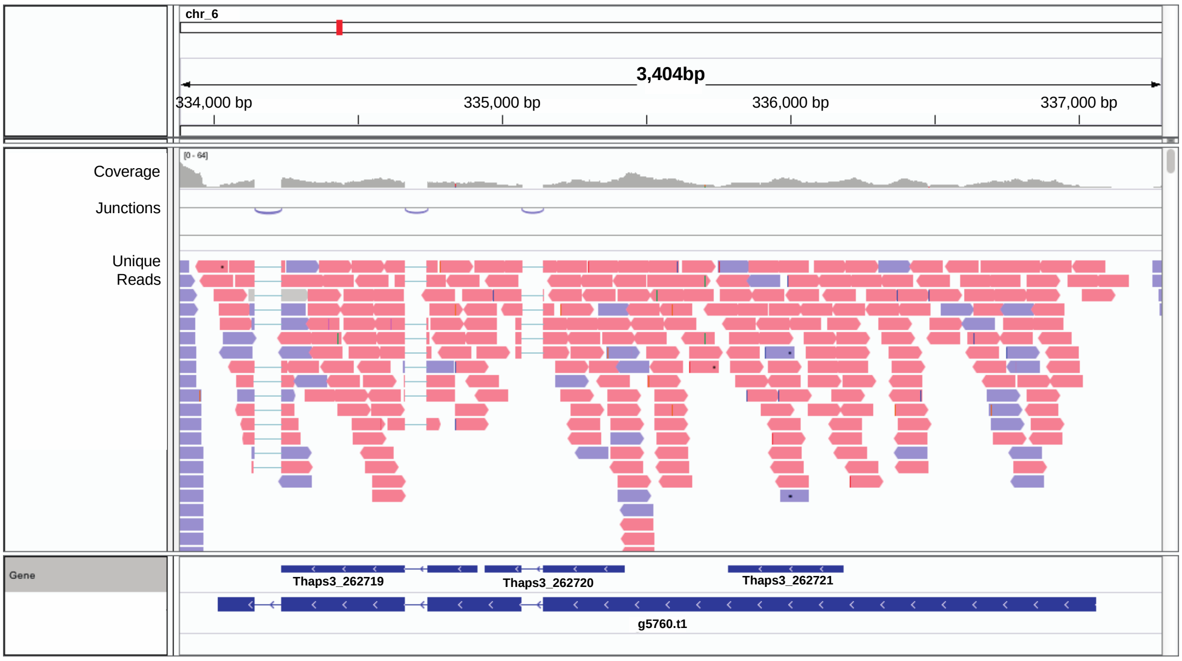

(b)

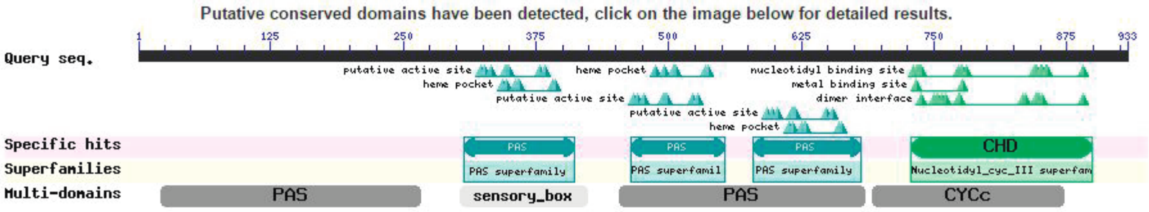

Fig. S5 PAS Cyclase Gene Model screenshot from Integrated Genomics Viewer (IGV) showing updated gene model for a putative PAS-Cyclase (a) and a screenshot from NCBI BlastP showing conserved domains (b). Thaps3 gene models (Thaps3_262719, Thaps3_262720, and Thaps3_262721) are combined into a single gene model.

New Phytologist

Table S1 Summary of silicon starvation experiments conducted and parameters quantified

Exp.	Cell Counts	Lipid Dye	Instrument	FAME Analysis	HPLC Pigments	Cell Cycle	RNA	Pvs. E	FRRf	Chloroplast Analysis
Si- #1	--	Nile Red	Spectrophotometer	--	--	--	--	--	--	--
Si- #2	--	Nile Red	Spectrophotometer	--	--	--	--	--	--	--
Si- #3	--	Nile Red	Spectrophotometer	--	--	--	Microarray	--	--	--
Si- #4	--	Nile Red	Spectrophotometer	--	--	--	--	--	--	--
Si- #5	x	Bodipy	Imaging Flow Cytometer	--	x	x	--	--	--	--
Si- #6	x	Bodipy	Imaging Flow Cytometer	--	x	x	--	--	--	--
Si- #7	x	Bodipy	Imaging Flow Cytometer	x	x	--	--	x	x	--
Si- #8	x	--	Imaging Flow Cytometer	--	--	--	--	--	--	x
Si- #9	x	Bodipy	Imaging Flow Cytometer	x	--	x	RNA-Seq	--	--	--
Si- #10	x	Bodipy	Imaging Flow Cytometer	x	--	x	RNA-Seq	--	--	--

Methods S1 Detailed methods.

Cell counts and cell cycle analysis

Cell concentrations were determined by Neubauer hemocytometer with a minimum of 200 cells counted per sample. For cell cycle stage determination, 12.5 ml of experimental culture was harvested by centrifugation (6 min at 4,000 ***g***), extracted in 12ml of 100% ice-cold methanol, and kept at 4°C until analysis. Cells were pelleted, and washed 3X with Tris-EDTA buffer (TE, pH 8.0), and then re-suspended in 1ml TE to treat with RNase A (0.3 mg ml^{-1}) at 37°C for 40 minutes. Cells were stained with SYBR® Green I (10,000X, Life Technologies, Carlsbad CA, USA) at a 1X final concentration from a 100X working stock made in dimethyl sulfoxide (DMSO), for ≥ 10 minutes and kept in the dark on ice until analysis within 4 hours. Cells were analyzed using the Becton Dickinson Influx sorting cytometer (BD Biosciences, San Jose, CA, USA). Data were analyzed using FlowJo cytometry software (Tree Star Inc. Ashland, OR, USA).

Lipid content with Nile Red and BODIPY

At each time point, triplicate samples (10 ml each) were sampled from the experimental cultures and pelleted by centrifugation (6 min at 3,200 ***g***). Pellets were kept frozen at -20°C until analysis. For Nile Red analysis, each pellet was re-suspended in 1ml 2.3% NaCl solution and stained with 6.25 µl Nile Red (Life Technologies) working stock (12.5 mg l^{-1} in acetone). Samples were incubated in the dark for 20-30 min before reading 200 µl in triplicate on a microtiter plate reader at exc/em: 485/576 nm. For BODIPY analysis, each pellet was re-suspended in 500 µl 2.3% NaCl and stained with a final concentration of 2.6 µg ml^{-1} of a 1 mg ml^{-1} BODIPY stock dissolved in DMSO (BODIPY 493/503, Life Technologies). Samples were incubated on ice for a minimum of 10 min and a maximum of 4 hr BODIPY fluorescence was quantified using imaging flow cytometry (ImageStreamX, Amnis Corp., Seattle, WA, USA) and interrogated with a 488 nm laser. Post-acquisition spectral compensation and data analysis were performed using IDEAS software (Amnis Corp.). Fluorescence intensity was determined for a minimum of 5,000 cells.

Fatty acid methyl ester (FAME) analysis

For lipid analysis, approximately 800 ml of culture (at a concentration of *c*. 5 × 10^5 cells ml^{-1}) was harvested by centrifugation and rinsed with 0.4M ammonium formate. Cells were placed in a glass tube under N_2 gas and stored at -80° C until analysis. Before analysis, cells were lyophilized overnight and the amount of material submitted to the extraction was accurately weighed (between 5–13 mg). Lipids were extracted following the Folch *et al.* (1957) method. First, lipid extracts were homogenized with a 2 : 1 chloroform : methanol (v : v) with 0.01% of BHT (butylated hydroxytoluene) solvent mixture and ultrasonicated for 20 min in an ice bath. The mixture was then washed with a saline solution of 0.88% KCl.

Fatty acids in the extracts were determined by transmethylation with 14% boron trifluoride/methanol at 70°C. The concentrations of the fatty acid methyl esters (FAME) were determined with a Hewlett Packard 5890 Series II gas chromatograph equipped with a flame ionization detector (GC/MS-FID; Dodds *et al.*, 2005). Separation was achieved in a DB-Wax column (30 m, 0.25 mm, and 0.25 µm) with a helium carrier gas at a flow of 1.4 ml min^{-1}. The injector, FID, and MS sources temperatures were 220, 300, and 230°C, respectively. Samples (1 µl) were injected with a split ratio of 50 : 1. FAMEs were identified by comparison of retention times to authentic lipid standards (68A and 68D, Nu-Check Prep, USA). Quantification of C14 to C18 and C20 to C24 fatty acids was performed relative to known concentrations and Flame Ionization Detection (FID) peak areas of C13 and C19 FAME internal standards, respectively. The response factor for each standard compound (in GLC 68A and 68D) of interest was applied to the respective sample peak areas to calculate the final concentration of each peak of interest. The fatty acid methylation reaction (i.e. transesterification) efficiency was determined from the C15 FA internal standard added to each lyophilized algal pellet. Each sample was analyzed in duplicate.

Pigment analysis

Culture samples (10 ml) were collected and immediately filtered through GF/F Whatman filters (25 mm) and stored at -80°C until their analysis by HPLC. Filters were extracted in 90% HPLC-grade acetone and ultrasonicated in an ice bath for 10 min, stored at -20°C for 24 h and

prefiltered through a 0.45 µm Whatman nylon Puradisk filter before injection. Afterwards, pigments were separated using a reverse phase C-8 column following the method of Zapata *et al.* (2000). External pigment standards were used for system calibration (DHI, Denmark). Samples were analyzed on a Waters 600 HPLC system, equipped with a Thermo Separation Products AS3000 sampler, a TSP Spectra 100 variable wavelength (fixed at 440 nm), a Waters 996 diode array (scanning 330–800 nm) and a Waters 470 scanning fluorescence detector. Data were collected and analyzed using the Waters Millennium 32 software package. Each sample was analyzed in duplicate.

Transcriptomics

RNA isolation For experiments Si- #3, Si- #9, and Si- #10, 750 ml of culture was removed, treated with cycloheximide (final concentration, 20 µg ml^{-1}) and harvested by filtration. RNA isolated from 6 time points (0, 4, 8, 12, 18, and 24 h). Cells were pelleted and stored at -80°C prior to total RNA isolation (Hildebrand & Dahlin, 2000). RNA from experiment Si- #3 was processed for hybridization to an Affymetrix GeneChip whole genome tiling array while RNA from experiments Si- #9 and Si- #10 were processed for Illumina-based RNA-Seq.

Microarray hybridization and processing The Affymetrix microarray was designed and analyzed at the gene and exon level with a total of 524,909 sense strand probes (average of 16 probes per gene), based on gene model predicted transcripts for *T. pseudonana,* version 3.0 (http://genome.jgi-psf.org/Thaps3/Thaps3.home.html). Included on the array were 33,886 antigenomic probes to account for nonspecific hybridization. For each hybridization, double stranded cDNA was synthesized from 7 µg of total RNA with no amplification using the GeneChip® WT Amplified Double-Stranded cDNA Synthesis Kit (Affymetrix, Santa Clara, CA). Cleanup of double-stranded cDNA was done with the GeneChip® Sample Cleanup Module (Affymetrix). Fragmentation and end-labeling was performed using the GeneChip® WT Double-stranded DNA Terminal Labeling Kit (Affymetrix). Hybridization of labeled targets on the arrays was carried out using the GeneChip® Hybridization, Wash, and Stain Kit (Affymetrix). The arrays were then scanned with the GeneChip® Scanner, to generate the probe cell intensity data files.

Initial data analysis was performed as described in Shrestha *et al.* (2012). Microarray data are presented in Table S2.

Initial analysis of gene level expression was performed using Affymetrix Expression Console Software, version 1.1. The Robust Multichip Analysis (RMA) algorithm was applied to the probe cell intensity data files for all experimental conditions, using default parameters in the RMA-sketch workflow for core gene level analysis. Hybridization results were visually inspected for outliers using the Affymetrix Expression Console quality control metrics. Log base two normalized intensities were then exported based on probe set identifiers. The probe set identifiers in the summarization files were mapped to the filtered gene model predicted transcript identifiers for *T. pseudonana*, version 3.0. Gene annotation mapping for the transcripts was obtained from a database created and maintained at the J. Craig Venter Institute. The annotations were appended to the intensity summarization files and converted to tab-delimited text files.

Normalized and mapped probe intensities were then subjected to a two-pronged test for differential expression which included a false discovery rate (FDR) corrected student *t*-test statistic along with a log fold change cutoff of +/- 1. Two tailed unpaired t-tests were performed between time-points using the standard function within Microsoft Excel. The FDR correction was performed via the method of Benjamini & Hochberg (1995) implemented by the R statistical software package. Fold changes were calculated as the difference of any time-point from the initial 0hr time-point. Genes were considered to be differentially expressed if they had an FDR corrected p-value < .05 and a fold change greater or less than ± 1.

RNA-Seq sequencing and processing Before library preparation, each RNA sample was subjected to quality control evaluation as follows. The concentration and purity of the RNA samples were assayed by a NanoDrop spectrophotometer (Thermo Scientific, Waltham, MA, USA), with a required A_{260}/A_{280} ratio between 2.0 and 2.2, and an A_{260}/A_{230} ratio above 2.0. If RNA samples failed to pass the A_{260}/A_{230} ratio test, they were subjected to an additional precipitation to remove contaminants. RNA quality was evaluated with a BioAnalyzer (Agilent

Technologies, Santa Clara, CA, USA) on an Agilent RNA 6000 Nano chip following the manufacturer's instructions. RNA integrity was quantified by Agilent 2100 Expert software.

Next, cDNA libraries were prepared from 4 µg of total RNA for each sample using the Illumina TruSeq Stranded mRNA Sample Prep kit (Illumina, San Diego, CA) according to manufacturer's protocols (Rev. D), which includes a poly-A purification step. The resulting libraries were evaluated for size on an Agilent DNA High Sensitivity chip following the manufacturer's instructions, and quantified by quantitative PCR according to Illumina's protocols. Each library was diluted to 2 nM with 10 mM Tris-Cl, 0.1% Tween 20. Bar-coded libraries were then pooled (10 or 11 per pool), and sequenced on an Illumina HiSeq 2000 with 100 nt paired end reads.

Raw reads were demultiplexed and quality trimmed and all reads mapped to the *T. pseudonana* genome version 3 exons (Thaps3 genome downloaded from JGI) using TopHat2 (Kim et al. 2013). For RNA-Seq data, differential expression was calculated using DESeq2 (Love et al. 2014). RNA-Seq data are presented in Table S2.

Accessibility Transcriptomic data are available at NCBI's Gene Expression Omnibus under GEO series accessions GSE75651 (microarray) and GSE75460 (RNA-Seq).

Photosynthesis vs irradiance

Photosynthetic characteristics were obtained from photosynthesis–irradiance (P–I) measurements using a modified ^{14}C-bicarbonate incorporation technique (Lewis & Smith, 1983; Arrigo *et al.*, 1999). P-I incubations were carried out in a photosynthetron incubator at 24 irradiances ranging from 0 to 830 µmol photons m^{-2} s^{-1}. Illumination was provided by two 150W tungsten-halogen lamps and adjusted within each vial chamber by neutral density filters. Total photosynthetically available radiation (PAR, 400–700 nm) within each illumination chamber was measured using a Biospherical Instrument QSL 101 sensor. P-I experiments were carried out at 18°C.

For each P–I curve, a 60 ml sample of culture was spiked with 0.06 mCi $NaH^{14}CO_3$ to obtain a final activity of 0.001 mCi mL^{-1}. The spiked sample was distributed in 2 ml aliquots to 7

ml glass scintillation vials. Twenty-four of these vials were placed in the individually illuminated chambers within the incubator and incubated for 1 h. Two vials (time-zero samples) were acidified immediately with 200 µl of 20% HCl and placed in a hood during the 24 h experiment. Total activity in the samples was determined by adding 100 µL of sample at 0 h into two scintillation vials containing 100 µl of 1M NaOH. Five milliliters of liquid scintillation cocktail (ECOLUME™) was then added.

After incubation, the samples were acidified with 200 µL of 20% HCl and placed in a hood for 24 h to drive off unincorporated inorganic radioisotope. After 24 h ventilation, all samples received 5 ml of ECOLUME™ liquid scintillation cocktail and were vigorously shaken.

Carbon uptake, normalized by Chl *a*, was calculated from radioisotope incorporation. The photosynthetic response was modeled by curve fitting as suggested by Platt *et al.* (1975):

$$P^B = P_{max}^B \tanh(\alpha * E / P_{max}^B)$$

Where P^B is Photosynthesis per unit biomass (Chl *a*) in units mgC mgChla^{-1} h^{-1}, P_{max}^B is maximum rate of photosynthesis per unit of biomass, α is the initial slope in units of [mgC mgChla^{-1} h^{-1} (µmol photons m^{-2} s^{-1})$^{-1}$] and E is irradiance in units of µmol photons m^{-2} s^{-1} .

Fast repetition rate fluorescence

The photochemical quantum yield of photosystem II (F_v/F_m) and the functional absorption cross section (σ_{PSII}) of photosystem II (PSII) was measured using a Chelsea Fastracka Fast Repetition Rate Fluorometer, programmed to deliver single turnover saturation of PSII from 100 flashlets of 1.1 µs at 1 µs intervals. Samples were dark acclimated for >30 min, after which single turnover fluorescence induction curves were measured over a range of background light levels. Photosynthetic parameters (F_v/F_m and σ_{PSII}) were estimated by fitting standard models to data to determine values of F_o (initial fluorescence), F_m (maximal fluorescence), F_v/F_m (= (F_m-F_o)/F_m), and σ_{PSII} (Kolber *et al.*, 1998).

New Phytologist

Imaging flow cytometry for chloroplast replication analysis

At each time point, samples (10 ml each) were sampled from the experimental culture and pelleted by centrifugation (6 min at 3,200 ***g***). Pellets were frozen at -20°C until analysis (<1 month). Pellets were resuspended in 500 µl 2.3% NaCl solution and analyzed using imaging flow cytometry as previously described. Post-acquisition data analysis was performed using IDEAS software using a minimum of 2000 cells (Amnis Corp.). Chloroplasts were quantified by creating a custom spot count feature in IDEAS.

REFERENCES

Arrigo KR, Robinson DH, Worthen DL, Dunbar RB, DiTullio GR, VanWoert M, Lizotte MP. 1999. Phytoplankton community structure and the drawdown of nutrients and CO_2 in the Southern Ocean. *Science* **283:** 365-367.

Benjamini Y, Hochberg Y. 1995. Controlling the False Discovery Rate: A practical and powerful approach to multiple testing. *Journal of the Royal Statistical Society. Series B (Methodological)* **57:** 289-300.

Dodds ED, McCoy MR, Rea LD, Kennish JM. 2005. Gas chromatographic quantification of fatty acid methyl esters: Flame ionization detection vs. electron impact mass spectrometry. *Lipids* **4:** 419-428.

Folch J, Lees M, Sloane Stanley GH. 1957. A simple method for the isolation and purification of total lipids from animal tissues. *The Journal of Biological Chemistry* **226:** 497-509.

Hennon GMM, Ashworth J, Groussman RD, Berthiaume C, Morales RL, Baliga NS, Orellana M, Armbrust EV. 2015. Diatom acclimation to elevated CO_2 via cAMP signalling and coordinated gene expression. *Nature Climate Change* **5:** 761-765.

Hildebrand M, Dahlin K. 2000. Nitrate transporter genes from the diatom *Cylindrotheca fusiformis* Bacillariophyceae: mRNA levels controlled by nitrogen source and by the cell cycle. *Journal of Phycology* **36:** 702-713.

Kim D, Pertea G, Trapnell C, Pimentel H, Kelley R, Salzberg, SL. 2013. TopHat2: Accurate alignment of transcriptomes in the presence of insertions, deletions and gene fusions. *Genome Biology* **14:** R36.

Kolber ZS, Prášil O, Falkowski PG. 1998. Measurements of variable chlorophyll fluorescence using fast repetition rate techniques: defining methodology and experimental protocols. *Biochimica et Biophysica Acta* **1367:** 88-106.

Lewis MR, Smith JC. 1983. A small volume short-incubation-time method for measurement of photosynthesis as a function of incident irradiance. *Marine Ecology Progress Series* **13:** 99-102.

Love MI, Huber W, Anders S. 2014. Moderated estimation of fold change and dispersion for RNA-seq data with DESeq2. *Genome Biology* **15:** 550.

Platt T, Denman KL, Jassby AD. 1975. The mathematical representation and prediction of phytoplankton productivity. *Fisheries and Marine Service Technical Reports* **523:** 110.

Shrestha RP, Tesson B, Norden-Krichmar T, Federowicz S, Hildebrand M, Allen AE. 2012. Whole transcriptome analysis of the silicon response of the diatom *Thalassiosira pseudonana. BMC Genomics* **13:** 499.

Zapata M, Rodríguez F, Garrido JL. 2000. Separation of chlorophylls and carotenoids from marine phytoplankton: A new HPLC method using a reversed phase C_8 column and pyridine-containing mobile phases. *Marine Ecology Progress Series* **195:** 29-45.
